# Enhancing the Photocatalytic Performance for Norfloxacin Degradation by Fabricating S-Scheme ZnO/BiOCl Heterojunction

**DOI:** 10.3390/molecules29194738

**Published:** 2024-10-07

**Authors:** Rongpeng Yang, Guang Lu, Hongyu Liang, Zheng Li, Jiling Liang, Zhen Chen

**Affiliations:** 1 School of Environmental & Safety Engineering, Liaoning Petrochemical University, Fushun 113001, China; 2 School of Civil Engineering, Liaoning Petrochemical University, Fushun 113001, China; luguang20121101@126.com (G.L.);; 3 School of Environmental and Chemical Engineering, Shenyang Ligong University, Shenyang 110159, China

**Keywords:** ZnO, BiOCl, heterojunction, photocatalytic degradation, NOR

## Abstract

Construction of S-scheme heterojunctions can effectively limit the recombination of photogenerated e^−^ and h^+^, thus improving photocatalytic activity. Therefore, S-scheme ZnO/BiOCl (molar ratio = 1:2) n–n heterojunctions were synthesized via a hydrothermal–hydrolysis combined method in this study. The physical and chemical properties of the ZnO/BiOCl heterojunctions were characterized by XRD, XPS, SEM, TEM, DRS, N_2_ adsorption–desorption and ESR. Additionally, the photoelectric performances of ZnO/BiOCl heterojunctions were investigated with TPR, M–S plot and EIS. The results show that photocatalytic degradation of NOR by ZnO/BiOCl reached to 94.4% under simulated sunlight, which was 3.7 and 1.6 times greater than that of ZnO and BiOCl, respectively. The enhanced photodegradation ability was attributed to the enhancement of the internal electric field between ZnO and BiOCl, facilitating the active separation of photogenerated electrons and holes. The radical capture experiments and ESR results illustrate that the contribution of reactive species was in descending order of ·OH > h^+^ > ·O_2_^−^ and a possible mechanism for the photodegradation of NOR over S-scheme ZnO/BiOCl heterojunctions was proposed.

## 1. Introduction

In the past few years, with the rapid development of industrialization and urbanization, antibiotic pollution of the environment has become increasingly serious [1], exposing aquatic organisms to chronic toxicity and leading to increased drug resistance in humans [2]. Norfloxacin (NOR), a third-generation 4-quinolone antibacterial agent, is commonly used in the treatment of enteric dysentery and is more difficult to oxidize than other organic pollutants like tetracycline hydrocholoride and organic dyes [3,4,5,6,7,8]. Many studies have shown that photocatalytic oxidation is a green and an effective way to solve this problem [9]. In addition, the ability of the catalyst to produce highly reactive radicals with low energy consumption and high efficiency is more beneficial than other methods in photocatalytic reactions [10].

In recent years, BiOCl has attracted attention as a semiconductor because of its unique two-dimensional layered structure, which consists of two different layers of Cl^−^ and [Bi_2_O_2_]^2+^ arranged alternately along the c-axis. Its unique structure in combination with its strong internal electrostatic field could efficiently separate the photogenerated electron and hole pairs, and thus improve the photocatalytic performance [11,12,13,14]. However, pure BiOCl suffers from low photocatalytic performance due to the rapid recombination of electron–hole pairs, small surface area and its broad prohibition [15,16,17]. To solve these problems, various techniques have been proposed, such as element doping, defect engineering, morphology modification and heterojunction construction [18]. Among these, compositing BiOCl with another semiconductor that has a suitable band structure by which to construct a heterojunction is considered to be a promising technology [19]. For instance, Qian et al. prepared BiVO_4_/BiOCl with a core-shell structure and the degradation of CIP, BHA, phenol, bisphenol A and cumene glycosides after irradiation with visible and near-infrared light for 180 min was in the range of 49.36% to 71.32% and 37.88% to 62.86%, respectively [2]. Chang et al. synthesized BiOCl/ZnO p–n heterojunctions by a solvothermal method and the photocatalytic activities of RhB was 100% under UV–vis light irradiation [14].

In particular, the step-scheme (S-scheme) heterojunction has garnered considerable recognition because of the internal electric field formed by the electrons’ spontaneous migration from reduction photocatalyst (RP) to oxide photocatalyst (OP), which could effectively inhibit the recombination of photogenerated carriers and produce the efficient redox property [20]. Zinc oxide (ZnO) is a well-known semiconductor due to its abundant reserves, stable chemical properties and high electron mobility [21]. Importantly, it is an n-type semiconductor with suitable CB and VB band positions [22] that can act as an oxidizing photocatalyst and can combine with BiOCl to form an S-scheme heterojunction. Many ZnO/BiOCl p–n heterojunctions have been reported to be successfully prepared via the hydrothermal method with low output, technological complexity and high energy consumption, resulting in the high price of catalyst preparation. Yang et al. prepared a BiOCl/ZnO p–n heterojunction with a unique semi-coherent interface and showed excellent catalytic performance and outstanding stability in the photodegradation of RhB, tetracycline and ciprofloxaci [23]. However, the ZnO/BiOCl p–n heterojunctions still have some drawbacks, including a poorly matched interface, poor stability, and a complex fabrication strategy [23,24]. Compared with the ZnO/BiOCl p–n heterojunction, the ZnO/BiOCl n–n heterojunction has an entirely different charge transfer route, which occurs through the recombination of the closer electron of ZnO and the hole of BiOCl [24]. This inhibits charge binding while maintaining a good redox capacity of the components [25], resulting in n–n heterojunctions that tend to have better photocatalytic properties and stability than p–n heterojunctions [26,27]. Consequently, the objective of this research is to prepare the ZnO/BiOCl n–n heterojunctions in order to remove NOR under simulated sunlight irradiation with an efficient photocatalytic activity via an easily operated, high-yield, energy-efficient and low-cost method. 

In this study, S-scheme ZnO/BiOCl heterojunctions were successfully prepared with a combined hydrothermal–hydrolysis method for the photocatalytic degradation of NOR. Consequently, ZnO/BiOCl heterojunctions were found to demonstrate superior photodegradation of NOR under simulated sunlight irradiation in comparison with blank ZnO and BiOCl, which can be primarily attributed to the enhanced photogenerated carrier separation in the S-scheme heterojunction of ZnO/BiOCl materials. Furthermore, the possible mechanism of NOR photodegradation by ZnO/BiOCl was proposed using free radical trapping experiments and electron spin resonance (ESR) tests.

## 2. Experimental

### 2.1. Chemicals and Material Preparation

#### 2.1.1. Chemicals

All the regents for synthesis and analysis were analytic grade and commercially available. Norfloxacin (C_16_H_18_FN_3_O_3_, NOR) was purchased from Macklin. Zinc nitrate hexahydrate (Zn(NO_3_)_2_·6H_2_O), hydrazine hydrate (N_2_H_4_·H_2_O), sodium hydroxide (NaOH), bismuth nitrate pentahydrate (Bi(NO_3_)_3_·5H_2_O), acetic acid (CH_3_COOH), potassium chloride (KCl) and ethanol (CH_3_CH_2_OH) were purchased from Sinopharm Chemical Reagent Co., Ltd. (Shanghai, China).

#### 2.1.2. Preparation of ZnO

ZnO was prepared by a hydrothermal method. Typically, 0.38 g Zn(NO_3_)_2_·6H_2_O was added to 9.4 mL N_2_H_4_·H_2_O (85%) to obtain mixture A. Then, mixture A was introduced to 60 mL 0.25 mol·L^−1^ NaOH solution under constant stirring for 60 min. Next, the resulting homogeneous suspension was placed into a Teflon lined stainless steel autoclave and heated at 90 °C for 5 h. Subsequently, the white solid powder was obtained after being washed several times and dried at 100 °C for 12 h. The resulting product was marked as ZnO. 

#### 2.1.3. Preparation of BiOCl

BiOCl was prepared according to a typical one-step solvothermal method [22]. Firstly, 4 mmol Bi(NO_3_)_3_·5H_2_O was added to 100 mL 0.5 mol·L^−1^ CH_3_COOH solution under stirring for 30 min, which was marked as mixture A. An amount of 4 mmol KCl was dissolved in 60 mL deionized water with stirring for 30 min to obtain mixture B. Secondly, mixture B was added drop by drop to mixture A. The resulting mixture was placed in a Teflon-lined autoclave and stored at 160 °C for 12 h. The white BiOCl powder was obtained after the resulting product was centrifuged, washed with deionized water and ethanol, and dried at 100 °C for 12 h.

#### 2.1.4. Preparation of ZnO/BiOCl Composite

ZnO/BiOCl composite samples were prepared by combined hydrothermal–hydrolysis method. Firstly, 1.455 g of Bi(NO_3_)_3_·5H_2_O was added to an acetic acid solution (100 mL H_2_O, 5 mL CH_3_COOH). Secondly, 0.122 g of as-prepared ZnO materials were added into the above solution and stirred at room temperature for 30 min until no ZnO powder was precipitated. Thirdly, 30 mL of KCl solution (0.0125 mol/L) was added quickly into the above mixed solution. After stirring for 30 min at room temperature, the suspension was allowed to stand for 3 h. Finally, the resulting precipitate was washed with deionized water and anhydrous ethanol for three times and dried at 100 °C for 12 h. The obtained solid (molar ratio = 1:2) was expressed as ZnO/BiOCl.

### 2.2. Characterization 

The crystal structures of catalysts were obtained by employing an X-ray diffractometer (XRD) by Cu-Kα radiation (λ = 0.15406 nm) under 40 kV and 150 mA with 5 °/min from 5° to 90°. Scanning electron microscopy (SEM) with an energy dispersive spectrometer (EDS) and elemental mapping and transmission electron microscopy (TEM) were performed to analysis the morphology and elemental composition of samples. The Brunauer–Emmett–Teller (BET) surface areas and the pore size distribution of prepared catalysts were identified by N_2_ adsorption–desorption on Autosorb-iQ2. The chemical composition and surface state were verified using X-ray photoelectron spectroscopy (XPS, using a monochromatic Al K X-ray source) and spectral deconvolution was performed using Avantage software (https://www.avantage.ca/en/, accessed on 1 October 2024). UV–vis absorption spectra (DRS) were determined to analyze the optical absorption properties of prepared samples. The electron spin resonance (ESR) was recorded to detect ·OH, h^+^ and ·O_2_^−^. In the ESR test, 5 mg catalyst was dissolved in 1 mL water or methanol for the detection of ·OH and ·O_2_^−^, respectively. Subsequently, 30 µL of the above solution was mixed with 100 mM of DMPO capture agent. For the detection of h^+^, the aqueous solution was mixed with 100mM of TEMP capture agent under the same conditions as described above.

### 2.3. The ZnO:BiOCl Ratio First Optimized

The photocatalytic activity of prepared samples was assessed by the degradation of NOR under simulated sunlight irradiation by a 500 W Xenon lamp. In each experiment, approximately 50 mg of photocatalyst was added to 200 mL of a 10 mg·L^−1^ NOR solution in special reactor with circulating cooling and a temperature sensor to keep the reaction at room temperature. Then, the above mixture was magnetically stirred for 30 min in the dark to achieve adsorption–desorption equilibrium before irradiation. During irradiation, approximately 5 mL of the suspension was removed every 30 min. The particles were removed by high-speed centrifugation and the clarified liquid was fetched for absorbance measurement. The concentration of NOR in the supernatant was detected using a UV–vis spectrophotometer (UV-1800PC, MAPADA, Shanghai, China) with the absorbance at 274 nm. A pseudo-first-order reaction kinetics model was used to describe the degradation kinetics of NOR [20,21,22]. The efficiency and kinetic constants (k) were studied using the following expressions, respectively:

(1) $$Efficiency=\frac{C_{t}}{C_{0}}\times 100\%\;$$

(2) $$-ln\left(\frac{C_{t}}{C_{0}}\right)=kt\;$$

where *C_t_* and *C*_0_ is the concentration of NOR at *t* and 0, respectively. In addition, *k* is the degradation rate constant.

In order to ensure the possible role of the main reactants of NOR on the ZnO/BiOCl heterojunctions in the solar degradation process, hole and radical trapping experiments were performed. In photocatalytic degradation experiments, isopropanol (IPA), benzoquinone (BQ), ethylenediaminetetraacetic acid disodium salt (EDTA-2Na) and silver nitrate (AgNO_3_) were employed as ·OH, ·O_2_^−^, h^+^ and e^−^ scavengers, respectively [28].

### 2.4. Photoelectrochemical Performance

The photoelectrochemical performance of as-prepared samples was studied using an electrochemical workstation (CHI 660, Chen Hua Instrument Company, Shanghai, China) with a three-electrode system. A platinum sheet, a saturated Ag/AgCl and the photocatalyst-prepared photoelectrode were used as counter electrode, reference electrode and working electrode, respectively. In addition, 0.3 M Na_2_SO_4_ was used as electrolyte for all of the tests. The working electrode was made from a sample of ITO-conducting glass (1 cm × 1 cm) drop coated with a slurry.

## 3. Results and Discussion

### 3.1. Structure, Composition and Morphology

**Figure 1:**
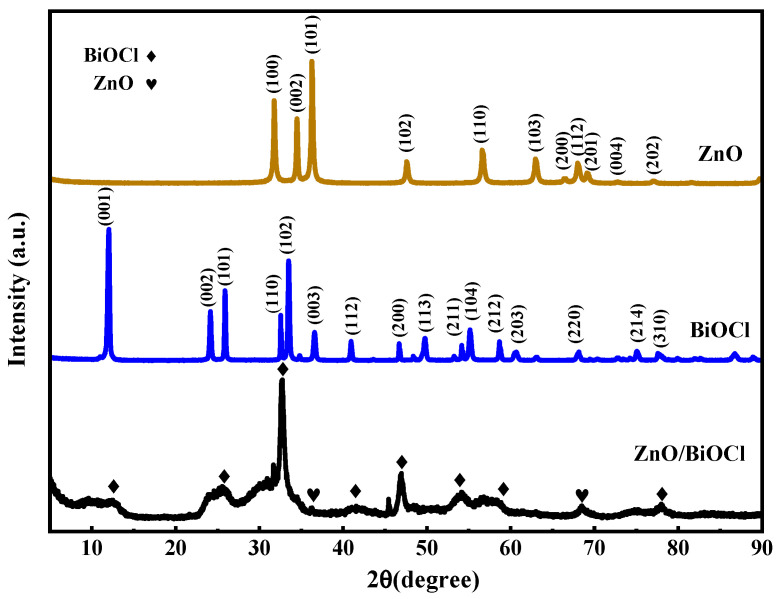
XRD patterns of the prepared samples.

The XRD patterns of pure BiOCl, pure ZnO and ZnO/BiOCl heterojunctions are shown in Figure 1. The characteristic peaks at 31.75°, 34.47°, 36.32°, 47.52°, 56.58°, 62.95°, 66.41°, 67.97°, 69.11°, 72.72° and 76.99° belong to (100), (002), (101), (102), (110), (103), (200), (112), (201), (004) and (202) diffraction planes, respectively, which are assigned to ZnO according to PDF#01-089-7102. Furthermore, the characteristic peaks at 12.04°, 24.16°, 25.90°, 32.55°, 33.49°, 36.58°, 40.94°, 46.68°, 49.77°, 54.15°, 55.13°, 58.65°, 60.66°, 68.13°, 75.07° and 77.59° correspond with the (001), (002), (101), (110), (102), (003), (112), (200), (113), (211), (104), (212), (203), (220), (214) and (310) structural planes of pure BiOCl (PDF#01-085-0861), respectively. The XRD spectra of the ZnO/BiOCl heterostructure is consisted of ZnO and BiOCl phases with no other impurity characteristic peaks, indicating the high purity of the obtained catalysts. In addition, the diffraction peaks of the ZnO/BiOCl have obvious changes in the strength of (110) peaks compared with standard peaks of pure BiOCl. This result suggests that the synthesized pure BiOCl samples possibly have the exposed crystal of (110) facets. And XRD diffraction peaks of the ZnO/BiOCl have an obviously vanishment in the intensity of (102) and (001) peaks compared with the standard peaks of pure BiOCl. This shows that the structure of ZnO/BiOCl has probably changed from that of BiOCl.

Significantly, it is clear from Figure 1 that the relative intensity of the (110) peak of ZnO/BiOCl has been greatly improved compared with BiOCl. This finding suggests that BiOCl in the composite samples develops with a preferred orientation of (110), producing a highly exposed (110) facet. This may be caused by the change in the structure of the ZnO/BiOCl heterojunction prepared by the combined hydrothermal–hydrolysis method, which is also verified later in the SEM and TEM characterizations. Zhang et al. developed BiOCl nanosheets exposed to (010), (001) and (110) facets and confirmed that BiOCl exposed to the (110) facet, when compared with the other samples, exhibited superior carrier separation performance, stronger interfacial carrier migration and higher photocurrent density in the experiments [29]. This suggests that the ZnO/BiOCl heterojunction prepared in this work may exhibit better photocatalytic activity when compared with other samples.

**Figure 2:**
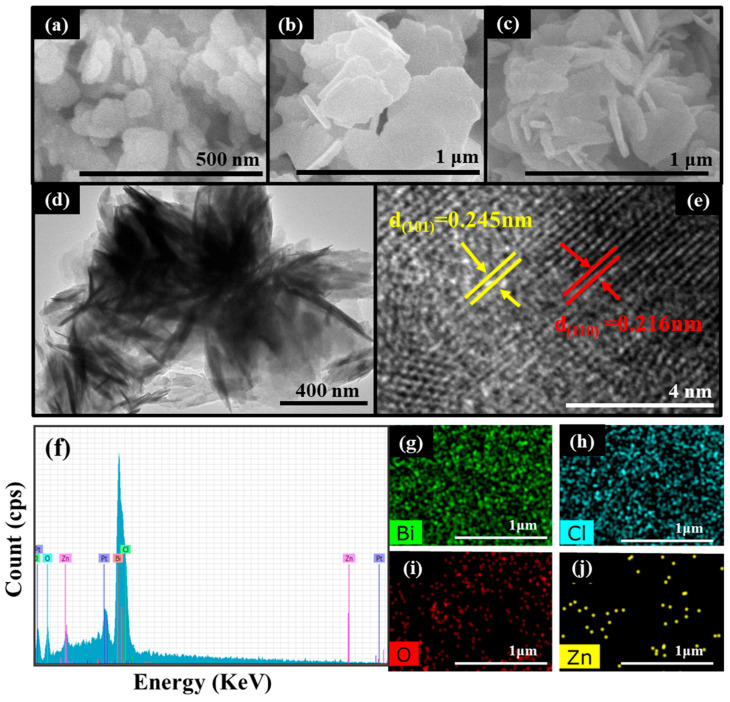
The SEM images of ZnO (**a**), BiOCl (**b**) and ZnO/BiOCl (**c**). TEM (**d**) and HRTEM (**e**) images of ZnO/BiOCl and EDS spectra of ZnO/BiOCl samples (**f**) and mapping images of Bi, Cl, O and Zn (**g**–**j**).

The morphology of ZnO, BiOCl and a ZnO/BiOCl composite are observed via the use of SEM. In the SEM test, all of the detected samples were treated with gold spraying. In Figure 2a, pure ZnO exhibits a 2D disc shape and the average particle size is approximately 50 nm. In Figure 2b, pure BiOCl displays a sheet structure with a diameter of around 60 nm. In Figure 2c, the ZnO/BiOCl composite exhibits a 3D laminar heterogeneous structure which is assembled from larger BiOCl flakes as well as ZnO nanoflakes attached to it with different orientations. While ZnO is grown in-situ on the surface of BiOCl, the size of ZnO increases significantly, indicating that the BiOCl has serious effects on the ZnO microstructure and contacts with ZnO to generate a homogeneous heterojunction.

In order to observe the distinct morphology and microstructure of the ZnO/BiOCl heterojunction, TEM and HRTEM studies were performed. As shown in the TEM image (Figure 2d), ZnO nanosheets (dark area) with different arrangements are grown perpendicularly on BiOCl nanosheets (gray area). According to HRTEM images (Figure 2e), the visible lattice patterns can be observed, indicating that both BiOCl and ZnO are successfully synthesized. Two lattice stripes with lattice spacing of 0.245 and 0.216 nm are apparent, belonging to the (101) crystallographic plane of ZnO and the (110) crystallographic plane of BiOCl. This strongly suggests that a heterojunction is formed between BiOCl and ZnO in our work, which favors charge separation and transfer in the interaction structure.

The energy dispersion spectroscopy (EDS) and elemental mapping of the ZnO/BiOCl catalyst are shown in Figure 2f–j. The elements of Zn, O, Bi and Cl can be observed over ZnO/BiOCl sample (Figure 2f). In addition, the mapping results prove that Zn, O, Bi and Cl elements distribute almost uniformly over the surface of the ZnO/BiOCl sample with 2.98%, 3.88%, 14.01% and 63.11% (Figure 2g–j), respectively.

**Figure 3:**
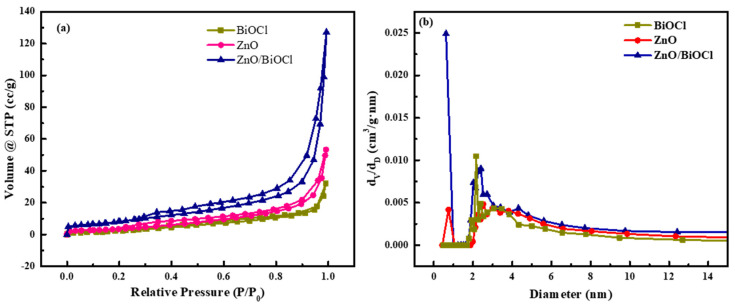
N_2_ adsorption–desorption isotherms (**a**) and pore size distribution (**b**) of ZnO, BiOCl and ZnO/BiOCl.

The specific surface area and porous structure of ZnO, BiOCl and ZnO/BiOCl were investigated by N_2_ adsorption–desorption tests. As show in Figure 3a, the N_2_ adsorption–desorption isotherms of the three catalysts show type-IV isotherms with H3 hysteresis loops. The isotherms of the ZnO and BiOCl are flat because of their small pore size and low specific surface area (as shown in Table 1). In addition, the pore size distribution reveals that the pore size assignments of ZnO, BiOCl and ZnO/BiOCl are all less than 12 nm and the pore sizes of ZnO/BiOCl are concentrated at 1 nm and 2~4 nm (as shown in Figure 3b).

The specific surface area, total pore volume and average pore size of ZnO, BiOCl and ZnO/BiOCl are shown in Table 1. The specific surface areas of ZnO, BiOCl and ZnO/BiOCl are 34.573, 21.646 and 115.912 m^2^/g, respectively, demonstrating that the introduction of BiOCl can increase the surface area of ZnO/BiOCl composites. As shown in Figure 2a–c, the structure of the ZnO/BiOCl heterojunction has changed compared with ZnO and BiOCl. In contrast with the layered structures of ZnO and BiOCl, ZnO and BiOCl in the ZnO/BiOCl heterojunction grow along different directions, which obviously increases the specific surface area of the heterojunction and provides more voids. Typically, the active sites exposed by photocatalysts become more numerous, owing to the increase in specific surface area, which improves photocatalytic activity [21].

### 3.2. XPS Analysis

**Figure 4:**
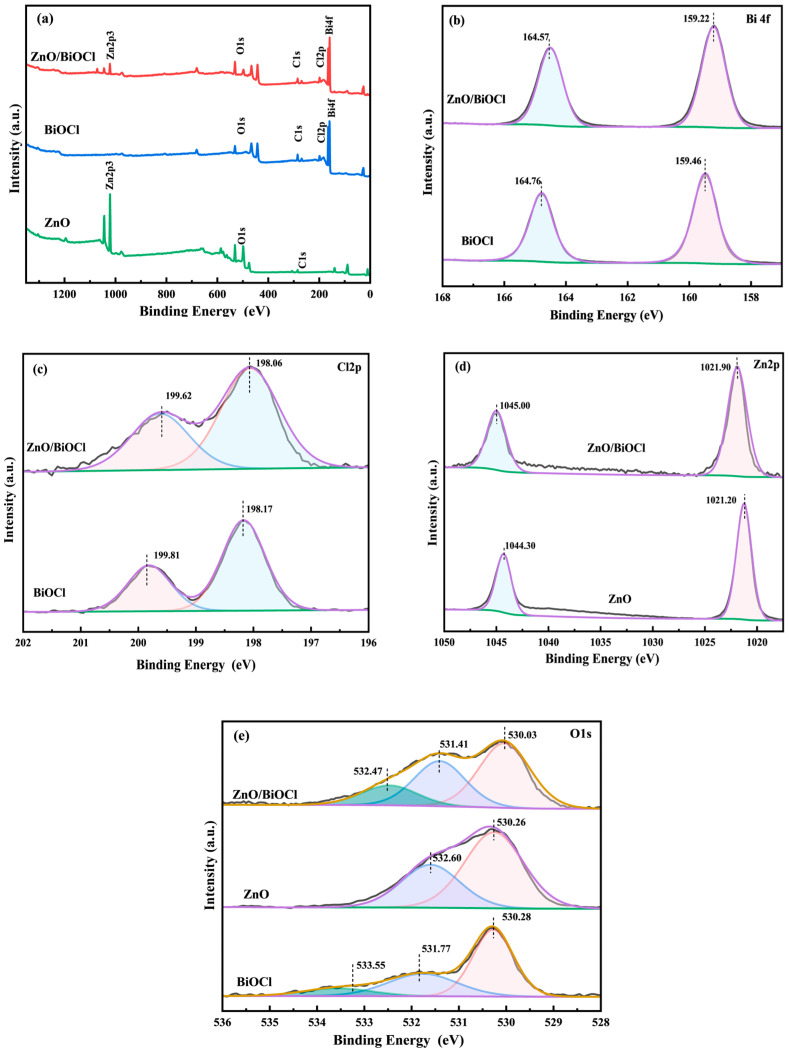
XPS spectra of pure BiOCl, pure ZnO and ZnO/BiOCl samples: full survey spectra (**a**), Bi 4f (**b**), Cl 2p (**c**), Zn 2p (**d**) and O 1s (**e**).

The surface elemental composition and atomic state of ZnO, BiOCl and ZnO/BiOCl were investigated by XPS (Figure 4). The total survey XPS spectrum (Figure 4a) of ZnO/BiOCl proves the existence of C, Zn O, Cl and Bi. Regarding the high-resolution XPS spectrum of Bi 4f (Figure 4b), two peaks at 159.46 and 164.76 eV on BiOCl are attributed to the Bi 4f_7/2_ and Bi 4f_5/2_ spin orbitals of Bi^3+^, respectively [18,19]. Regarding the high-resolution XPS spectra of Cl 2p (Figure 4c), two peaks at 198.17 and 199.81 eV over BiOCl can be attributed to the Cl 2p_3/2_ and Cl 2p_1/2_ spin orbitals of Cl^−^, respectively [11,18,19]. For the Zn 2p XPS spectrum (Figure 4d) of ZnO, two peaks at 1044.30 and 1021.20 eV are attributable to the +2 valence forms Zn 2p_1/2_ and Zn 2p_3/2_, respectively [10]. For O 1s (Figure 4e), the deconvoluted broadband profile identifies the contribution of three energy levels in the BiOCl sample. The peaks of BiOCl at lower (530.28 eV), intermediate (531.77 eV) and higher (533.55 eV) bands are assigned to the O^2−^ anion, the surface hydroxyl groups of the O 1 s and the adsorbed O_2_ or H_2_O molecules on the surface, respectively [12]. Furthermore, the deconvoluted broadband profile identifies the contribution of two energy levels in pure ZnO. The binding energies at 530.26 and 532.60 eV could correspond with the Zn–O and hydroxyls of the surface, respectively [10]. The O 1s signals of ZnO/BiOCl can be decomposed into three distinct peaks at 530.03, 531.41 and 532.47 eV, which are related to BiOCl, ZnO and adsorbed oxygen species at the surface and adventitious carbon-binding oxygen (C–O species), respectively [5,10,30,31,32]. 

As shown in Figure 4b,c, the energies of both the Bi 4f and Cl 2p peaks of ZnO/BiOCl are reduced compared with pure BiOCl, due to the migration of electrons from ZnO to BiOCl, which is in agreement with [18]. On the contrary, as shown in Figure 4d, the Zn 2p orbitals of ZnO/BiOCl migrate to higher binding energies, suggesting the loss of ZnO electrons. These results suggest that the strong interaction is formed in ZnO/BiOCl composite because of the intimate contact of ZnO with BiOCl [33]. This result further confirms the formation of heterojunctions in ZnO/BiOCl composite materials.

### 3.3. Optical Properties

**Figure 5:**
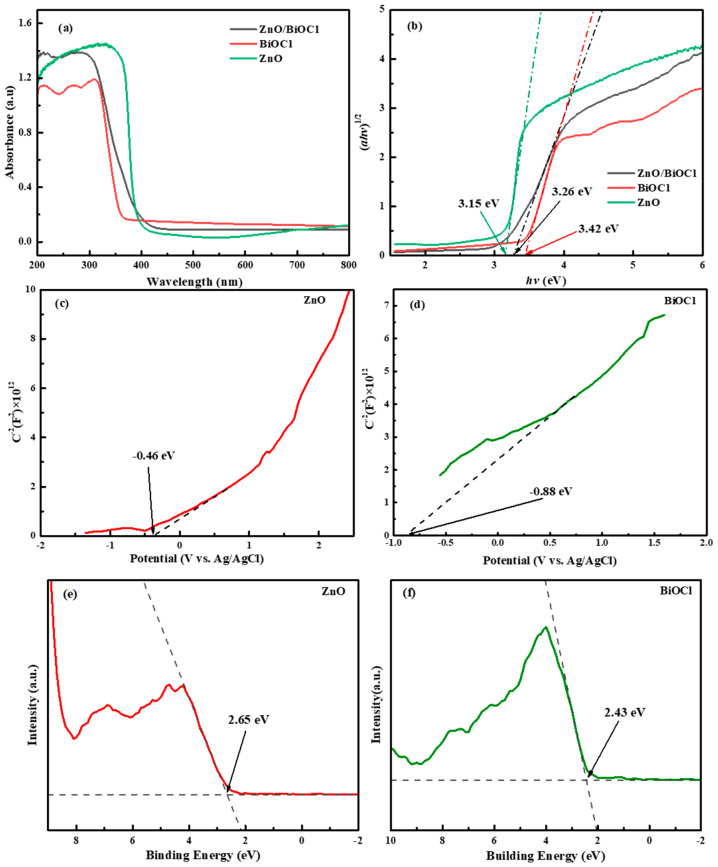
UV–vis diffuse reflectance spectra (**a**) and Tauc plots (**b**) of the prepared samples, Mott–Schottky plots of ZnO (**c**) and BiOCl (**d**), and XPS valence band spectra of ZnO (**e**) and BiOCl (**f**).

UV–vis DRS was employed to analyze the optical absorption of as-prepared samples. Figure 5a displays that all the materials show strong absorption of UV light in the wavelength range of 200–300 nm. The ZnO/BiOCl composite clearly enhances the absorption of UV light in the wavelength range of 200–300 nm over that of pure BiOCl [12]. As a result, the productivity of photon collection in composites under simulated sunlight irradiation can be enhanced.

The curve of (*αhν*)^1/2^ versus photon energy (*hν*) for the photocatalysts can be determined by the following equation:

*αhν* = *A*(*hν* − *Eg*)^n^ (3)

where *α*, *h*, *v*, *A* and *Eg* represent the absorption coefficient, plate constant, optical frequency, proportionality constant and bandgap energy, respectively. The value of n depends on the property of the semiconductor (*n* = 1/2 or 2 for direct bandgap or indirect bandgap separately) [34]. As shown in Figure 5b, the bandgap energy can be calculated from the tangent of the *hν* axis intercept of the extrapolated curve by considering the baseline. The bandgaps of BiOCl, ZnO/BiOCl and ZnO are 3.42, 3.26 and 3.15 eV, respectively. The results show that the bandgap energy is reduced after the composite of BiOCl and ZnO.

To confirm the conduction band potential (E_CB_), the Mott–Schottky equation was used to calculate the flat band potentials of BiOCl and ZnO [35]. As shown in Figure 5c,d, the intersection of the linear part of the MS curve with the *X* axis allows one to determine the values of −0.88 and −0.46 eV vs. Ag/AgCl for BiOCl and ZnO, respectively. Based on the Nernst equation, E_NHE_ = E_Ag/AgCl_ + 0.2 [36], the corresponding E_NHE_ are −0.68 and −0.26 eV vs. NHE for BiOCl and ZnO, respectively. According to the rule of thumb, the conduction band minimum position (E_CB_) of n-type photocatalysts is usually 0.2 eV more negative than E_fb_ [33,37]. Therefore, the E_CB_ of BiOCl and ZnO is thereby determined as −0.88 and −0.46 eV vs. NHE, respectively. The bandgap relationship of different samples with the bandgap relationship is as follows: valence band potential (E_VB_) = E_CB_ + E_g_ [35,36]. As a result, the E_VB_ of BiOCl and ZnO are 2.54 and 2.69 eV vs. NHE, respectively. 

To further research the accuracy of the energy band structure, XPS valence band spectra were employed to measure the valence band potentials (E_VB_, _XPS_). As shown in Figure 5e,f, the E_VB_, _XPS_ of BiOCl and ZnO are found to be 2.43 and 2.65 eV vs. NHE, respectively. The results are in good agreement with the calculated E_VB_. Based on the literature [3,38], the E_CB_ of n-type semiconductors is 0.1~0.3 eV higher than E_f_. In this study, E_f_ is set 0.2 eV lower than E_CB_. The E_g_, E_CB_, E_f_ and E_VB_ of ZnO and BiOCl are shown in Table 2.

### 3.4. Photoelectric Performance and Photocatalytic Activity

**Figure 6:**
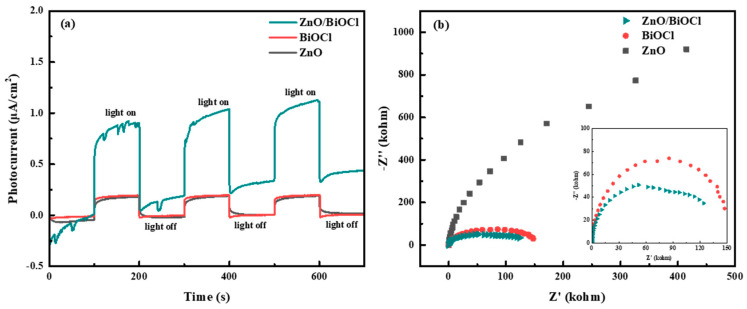
TPR (**a**) and EIS (**b**) of ZnO, BiOCl and ZnO/BiOCl.

The transient photocurrent response (TPR) and electrochemical impedance spectroscopy (EIS) property of the materials were investigated in order to reveal the efficiency of e^−^-h^+^ pair separation. As shown in Figure 6a, the current of all the samples increases rapidly under simulated sunlight irradiation and returns to the initial state after removing the light with good reproducibility, suggesting a good stability of their photocurrent response [39,40]. In addition, ZnO/BiOCl heterojunction shows the highest photocurrent response under simulated sunlight irradiation conditions, which indicates ZnO/BiOCl could produce a substantial amount of photogenerated carriers under simulated sunlight irradiation, with the highest effective separation and transition. In the EIS spectrum, the smaller arc radius reflects the smaller charge transfer resistance [12]. As shown in Figure 6b, the arc radius of ZnO/BiOCl is the smallest, suggesting that its charge transfer quality is best. These results verify that the ZnO/BiOCl heterojunction sample has higher separation rate and smaller transfer resistance, making it possible to have brilliant photochemical performance.

**Figure 7:**
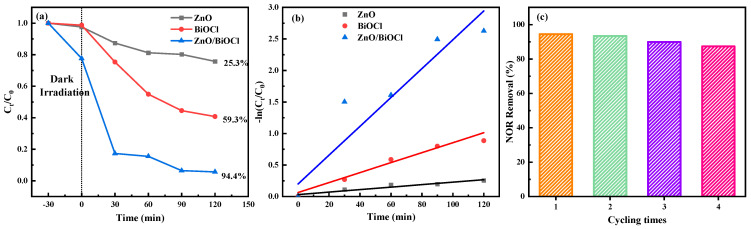
The photodegradation curves of NOR (**a**). First-order kinetic fitting curve (**b**) for different samples and cycling experiment of ZnO/BiOCl in NOR aqueous solution (**c**).

The photocatalytic degradation of NOR was studied under simulated sunlight irradiation at room temperature. As shown in Figure 7a, the degradation percentages of NOR by the ZnO, BiOCl and ZnO/BiOCl samples are 25.3%, 59.3% and 94.4% after 120 min, respectively. Clearly, the photocatalytic activity of the as-synthesized ZnO/BiOCl heterojunction can be effectively raised when compared with the ZnO or BiOCl. The enhancement in photocatalytic performance of ZnO/BiOCl catalysts can be attributed to (1) the construction of S-scheme heterojunctions between ZnO and BiOCl could sufficiently improve the separation and migration of electron-hole and to the way in which, (2) after adding ZnO to BiOCl, the ZnO/BiOCl catalyst possess a significantly increased specific surface area, resulting in the formation of more active sites. 

The photodegradation kinetics of the prepared ZnO/BiOCl, ZnO and BiOCl were further investigated using a pseudo-first-order kinetic equation (Equation (2)) based on the Langmuir–Hinshelwood model. The calculated rate constants (k) for the ZnO, BiOCl, and ZnO/BiOCl samples are 0.00198, 0.00791, and 0.02287 min^−1^, respectively, as shown in Figure 7b. The correlation coefficient (R^2^) for the ZnO, BiOCl, and ZnO/BiOCl samples are 0.9232, 0.9655 and 0.9584, respectively. The degradation rates are enhanced by about 11.55 and 2.89 times for ZnO/BiOCl than for ZnO and BiOCl, respectively. The comparison results of the oxidation data obtained on as-prepared catalysts with the catalysts presented in the literature are listed in Table 3. It can be found that the as-prepared ZnO/BiOCl heterojunction shows a better NOR degradation when compared with the other catalysts. However, the photocatalytic degradation of other antibiotics needs to be developed, which is the focus of our future work.

In addition, cyclic degradation of the ZnO/BiOCl photocatalyst was carried out to test the photocatalytic stability. As shown in Figure 7c, the degradation efficiency of NOR under sunlight irradiation decreases from 94.4% to 87.3% after four cycles, which proves that the ZnO/BiOCl catalyst structure has good recycling performance and certain practical application value. 

### 3.5. Photocatalytic Degradation Mechanism

**Figure 8:**
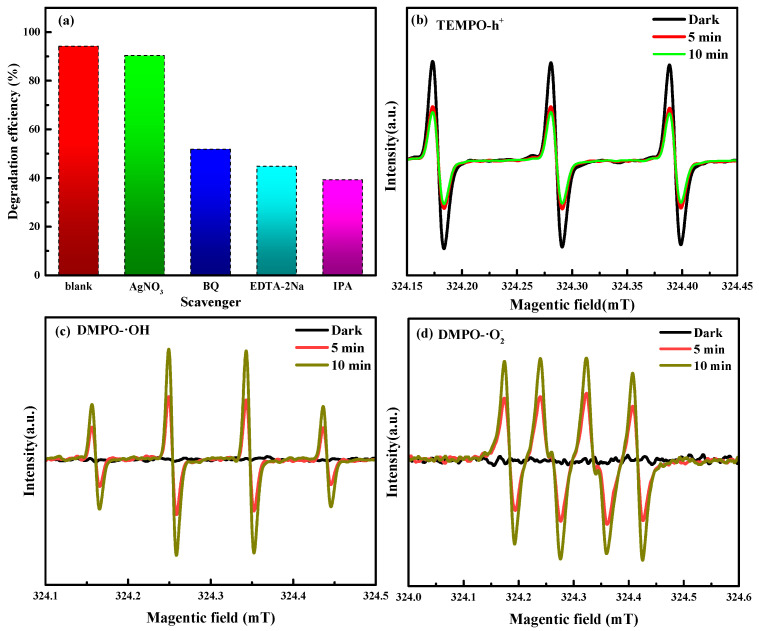
Free radical trapping experiments of ZnO/BiOCl (**a**), ESR spectra of TEMPO-h^+^ (**b**), DMPO-·OH (**c**) and DMPO-·O_2_^−^ (**d**).

To detect the reactive substances involved in the photocatalytic oxidation of NOR, comparative experiments were carried out on ZnO/BiOCl. Furthermore, BQ, AgNO_3_, EDTA-2Na and IPA were used to remove the superoxide radicals (·O_2_^−^), electrons (e^−^), holes (h^+^) and hydroxyl radicals (·OH) generated in the photocatalytic reaction. As shown in Figure 8a, the degradation efficiency of NOR is greatly inhibited by the addition of BQ, EDTA-2Na and IPA, reducing by 51.81%, 44.74% and 39.22%, respectively. In addition, the addition of AgNO_3_ only slightly affects the photocatalytic degradation of NOR. From the above results, it can be seen that ·O_2_^−^, h^+^ and ·OH are the primary reactive species in the photodegradation of NOR over the ZnO/BiOCl heterojunction. Moreover, ·OH plays a more important role than the others.

Electron spin resonance (ESR) analysis was further employed to monitor h^+^, ·O_2_^−^ and ·OH during the photocatalytic reaction. As shown in Figure 8b, the characteristic signal peaks of TEMPO-h^+^ are significantly weakened after irradiation when compared with those obtained in the dark conditions. However, the intensity of TEMPO-h^+^ peaks remain almost unchanged as the irradiation is extended from 5 min to 10 min. As shown in Figure 8c,d, no distinct characteristic peaks of DMPO-·O_2_^−^ or DMPO-·OH are observed under dark conditions, suggesting that the active species are only produced in the presence of light. Under simulated sunlight irradiation, characteristic signals with peak intensity ratios of 1:2:2:1 and 1:1:1:1 are detected, which belong to DMPO-·OH and DMPO-·O_2_^−^, respectively. With increase of irradiation, the characteristic signals of DMPO-·OH and DMPO-·O_2_^−^ are increased. These results indicate that the ZnO/BiOCl photocatalyst produces h^+^, ·OH, and ·O_2_^−^ radicals as it is exposed to sunlight, which is consistent with the results of the radical trapping experiments.

**Figure 9:**
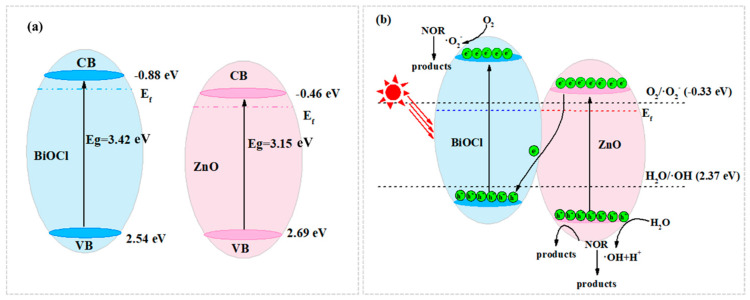
Schematic illustration of the band structure before (**a**) and after (**b**) contact of ZnO with BiOCl.

According to the above analysis of experiments, a viable photocatalytic mechanism for the degradation of NOR by ZnO/BiOCl composites was demonstrated, as shown in Figure 9. The ZnO/BiOCl heterojunction fulfills the criteria for the formation of S-type photocatalysts [41]. The CB site and Fermi level of BiOCl are both higher than those of ZnO (Figure 9a). When BiOCl and ZnO are in touch with each other, the electrons in BiOCl would be transferred to ZnO at the interface until the difference in E_f_ is zero [43]. Thus, the ZnO side would lose e^−^ and become electrically charged. The electrons are transferred from the CB of ZnO to the VB of BiOCl under simulated sunlight irradiation (Figure 9b). The potential on the ZnO side becomes positive due to the loss of electrons and, on the contrary, the potential on the BiOCl side becomes more negative due to the gain of electrons. The direction of electron transfer from ZnO to BiOCl is consistent with the shift in binding energy in the XPS experiments. The E_CB_ of BiOCl (−0.88 eV) is more negative than the E^θ^(O_2_/·O_2_^−^) = −0.33 eV, thus the enriched e^−^ on the CB of BiOCl could trap O_2_ to generate ·O_2_^−^ [44,45,46]. In addition, the remaining holes could actually become concentrated in the VB of BiOCl. The VB of ZnO (2.69 eV) is higher than E^θ^ (·OH/H_2_O) = 2.37 eV, thus h^+^ on VB of ZnO could oxidize H_2_O/OH^−^ to form ·OH. At the same time, h^+^ on VB of ZnO directly oxidizes NOR during the photocatalytic process [47]. Under simulated sunlight irradiation, the separation of e^−^ and h^+^ is effectively improved, thus the photocatalytic performance of the ZnO/BiOCl heterojunction is enhanced. Therefore, ·OH, ·O_2_^−^ and h^+^ are all essential reactive substances in the photodegradation of NOR and can degrade NOR into simpler aliphatic compounds and inorganic minerals, such as CO_2_, H_2_O and NH_4_^+^ [48], which can be described as follows:

(4) $$ZnO/BiOCl+h\nu \to e^{-}+h^{+}$$

(5) $$O^{2}+e^{-}\to \cdot O_{2}^{-}$$

(6) $$H_{2}O+h^{+}\to \cdot OH$$

(7) $$OR+\cdot O_{2}^{-}/\cdot OH\;/h^{+}\;\to H_{2}O+CO_{2}+products$$

## 4. Conclusions

In summary, S-scheme ZnO/BiOCl heterojunctions were successfully prepared by the hydrolysis process. With the loading of ZnO on BiOCl, the resulting ZnO/BiOCl composite provided effective separation and migration of the photogenerated charge carriers, as well as efficiently enhanced active sites, resulting in the photodegradation of NOR being higher than ZnO and BiOCl. Trapping experiments and ESR results reveal that the primary radical species in NOR photodegradation were ·OH, h^+^ and ·O_2_^−^. Overall, this work offers a novel, effective and stable photocatalytic nanocomposite for environmental containment removal.

## Figures and Tables

**Table 1:** BET surface area total, pore volumes and average pore size of prepared samples.

Sample	Surface Area (m^2^/g)	Total Pore Volumes (cc/g)	Average Pore Diameter (nm)
ZnO	34.573	0.089	2.518
BiOCl	21.646	0.054	2.158
ZnO/BiOCl	115.912	0.212	0.619

**Table 2:** E_g_, E_CB_, E_f_ and E_VB_ of ZnO and BiOCl.

Sample	E_g_ (eV)	E_CB_ vs. NHE (eV)	E_VB_ vs. NHE (eV)	E_f_ vs. NHE (eV)
ZnO	3.15	−0.46	2.69	−0.26
BiOCl	3.42	−0.88	2.54	−0.68

**Table 3:** Comparison table of the oxidation data.

Catalysts	Pollutants	Light Source	Reaction Time (min)	Removal Efficiency	References
BiVO_4_@BiOCl	TC	Vis/NIR	180	90.32%	[2]
g-C_3_N_4_ ZnO/BiOBr	Cr(Ⅵ)	UV light	180	96%	[14]
	MO	UV light	130	100%	
ZnO/Ag ZnO/Fe	Ibuprofen	Vis	120	86%	[21]
BiOCOOH/O-gC_3_N_4_	NOR	Vis	90	82%	[41]
BiOBr/ZnO	MO	Vis	120	76%	[42]
ZnO/BiOCl	NOR	Sunlight	120	94.40%	This work

## Data Availability

Data are contained within the article.
